# Protection of Human Colon Cells from Shiga Toxin by Plant-based Recombinant Secretory IgA

**DOI:** 10.1038/srep45843

**Published:** 2017-04-03

**Authors:** Katsuhiro Nakanishi, Shota Morikane, Shiori Ichikawa, Kohta Kurohane, Yasuo Niwa, Yoshihiro Akimoto, Sachie Matsubara, Hayato Kawakami, Hirokazu Kobayashi, Yasuyuki Imai

**Affiliations:** 1 Laboratory of Microbiology and Immunology, School of Pharmaceutical Sciences, University of Shizuoka, Shizuoka City, Shizuoka 422-8526, Japan; 2 Laboratory of Plant Molecular Improvement, Graduate Division of Nutritional and Environmental Sciences, University of Shizuoka, Shizuoka City, Shizuoka 422-8526, Japan; 3 Department of Anatomy, Kyorin University School of Medicine, Mitaka, Tokyo 181-8612, Japan; 4 Laboratory for Electron Microscopy, Kyorin University School of Medicine, Mitaka, Tokyo 181-8612, Japan

## Abstract

Shiga toxin is a major virulence factor of food-poisoning caused by *Escherichia coli* such as O157:H7. Secretory immunoglobulin (Ig) A (SIgA) is supposed to prevent infection of the mucosal surface and is a candidate agent for oral immunotherapy. We previously established a recombinant monoclonal antibody (mAb) consisting of variable regions from a mouse IgG mAb specific for the binding subunit of Shiga toxin 1 (Stx1) and the Fc region of mouse IgA. Here we produced a secretory form of the recombinant IgA (S-hyIgA) with transgenic *Arabidopsis thaliana* plant. All the S-hyIgA cDNAs (*heavy, light, J chain* and *secretory component*) were expressed under the control of a bidirectional promoter of a chlorophyll *a/b*-binding protein of *A. thaliana* without using a viral promoter. The plant-based S-hyIgA exhibited antigen binding, and was modified with plant-specific *N*-linked sugar chains. The Ig heavy chain and secretory components were observed in an intracellular protein body-like structure of the transgenic leaves on immuno-electron microscopy. An extract of the transgenic leaves neutralized the cytotoxicity of Stx1 toward butyrate-treated Caco-2 cells, a human colon carcinoma cell line. These results will contribute to the development of edible therapeutic antibodies such as those for the treatment of mucosal infection.

The intestinal mucosal surface is always exposed to many types of antigens derived from food, exogenous microbes and enteric bacteria. The invasion and potential harmful effects of these antigens on the body are blocked by mucosal immune systems including immunoglobulin A (IgA)^1^. IgA is the major antibody isotype on the mucosal surface, and it forms secretory IgA (SIgA). Two IgA monomers are linked through a joining (J) chain (termed dimeric IgA), followed by association with a secretory component (SC) that yields SIgA. SIgA is produced by two types of cells, IgA-secreting plasma cells and mucosal epithelial cells^2^. Plasma cells produce dimeric IgA and secrete it into the lamina propria. After binding to the polymeric immunoglobulin receptors (pIgR) on the epithelial cell membrane, the secreted dimeric IgA is transcytosed from the basolateral to the apical side. The extracellular domains of pIgR are cleaved by a protease to form SC. The dimeric IgA and SC complex is released into the intestinal lumen as SIgA. SIgA can function in a protease-rich environment such as the interior of the intestine because it is resistant to proteases^3^,^4^. SIgA is also important for protection of children from infection on oral feeding of breast milk containing SIgA^5^. In a porcine system, orally fed SIgA, but not IgG, was shown to protect piglets from infection of enterotoxigenic *Escherichia coli* (ETEC)^6^. Thus, SIgA specific for antigens on pathogens will be useful for treating mucosal infections based on the mechanism of oral passive immunity.

It is difficult to produce monoclonal SIgA using single mammalian cells because two types of cells are involved in nature. Although there have been several reports, the production of SIgA in a single mammalian cell is still challenging^7^,^8^. The first report of SIgA production using a plant expression system was earlier than that as to a mammalian cell system, and such reports on plant systems have recently been increasing^9^,^10^,^11^,^12^. A plant expression system has advantages as to oral passive immunity due to the ability of oral administration without or with minimal formulation when edible plant hosts are used^13^. In addition, such a system is more cost effective and exhibits higher scalability than mammalian ones for the production of therapeutic proteins^14^,^15^. Antibodies produced in a plant system are called plantibodies for short^14^. Thus, plant expression systems will be valuable for the production of SIgA and other agents aiming at oral passive immunity.

We previously established recombinant IgA antibodies aiming at oral passive immunization against Shiga toxin 1 (Stx1)^16^. Stx1 is responsible for food-poisoning caused by enterohemorrhagic *Escherichia coli* and *Shigella dysenteriae*^17^. Stx1 is an AB_5_ toxin consisting of a cytotoxic A subunit and a pentamer of cell-binding B subunits (Stx1B)^18^. Stx1B binds to the target cells via a cell surface glycolipid, globotriaosylceramide (Gb_3_). Gb_3_ is expressed on kidney, microvascular, leukocytic and neuronal cells, as well as many types of tumour cells^19^,^20^,^21^. Butyrate treatment of some cancer cell lines has been reported to induce expression of Gb_3_^22^. Because the cytotoxicity of Stx1 starts with the binding of Stx1B to cell surface Gb_3_, inhibition of the binding of Stx1B to Gb_3_ will be effective for preventing Stx1 toxicity. We established a recombinant antibody, termed hybrid-IgG/IgA (hyIgA for short), which has an amino acid sequence derived from a variable to the hinge region of the heavy chain as well as the light chain of mouse monoclonal IgG specific for Stx1B and the Fc region of the mouse IgA heavy chain. The dimeric hybrid-IgG/IgA expressed in Chinese hamster ovary cells together with mouse J chain efficiently neutralized Stx1 toxicity *in vitro.* The neutralizing activity of the dimeric one was stronger than that of the recombinant monomeric IgA and the original IgG mAb^23^,^24^. We then established transgenic *Arabidopsis thaliana* expressing the dimeric hybrid-IgG/IgA, and found that a leaf extract was capable of neutralizing Stx1 toxicity *in vitro*^25^. In this transgenic plant, the transcription of the *H* and *L chain* genes is controlled by a chlorophyll *a/b*-binding protein (CAB) promoter derived from *A. thaliana*, whereas the *J chain* gene is controlled by the cauliflower mosaic virus *35S* promoter. The *CAB* promoter is known to induce the co-expression of two proteins bi-directionally, and the expression level was reported to be high in leaf tissue^26^. In this study, we developed hybrid-IgG/IgA transgenic plants that express a secretory form of hybrid-IgG/IgA (S-hyIgA) only using the *CAB* promoter and terminators through a one-step transformation of four genes in a construct. We examined the protein assembly of the secretory form of IgA in leaf tissues and its neutralizing activity against Stx1 *in vitro*.

## Results

### Establishment of secretory hybrid-IgG/IgA transgenic* Arabidopsis thaliana*

**Figure 1:**
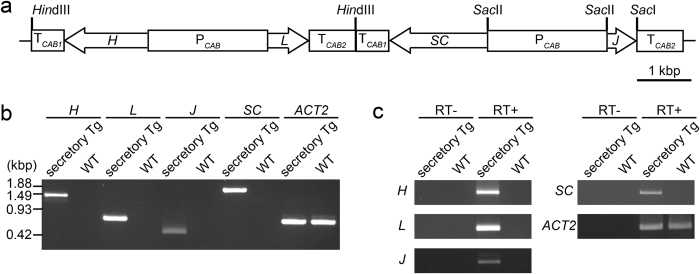
Production of secretory hybrid-IgG/IgA (S-hyIgA) transgenic *A. thaliana*. (**a**) Schematic diagram of S-hyIgA expression vector. P_*CAB*_, (2.2 kbp) chlorophyll *a/b*-binding protein promoter; T_*CAB1*_ (0.6 kbp) and T_*CAB2*_ (0.85 kbp), chlorophyll *a/b*-binding protein terminators; *H* (1.5 kbp), hybrid-IgG/IgA heavy chain; *L* (0.75 kbp), hybrid-IgG/IgA light chain; *J* (0.5 kbp), immunoglobulin joining chain; *SC* (1.8 kbp), secretory component. The total length of S-hyIgA expression vector is 26.4 kbp. (**b**) Detection of the *S-hyIgA* transgenes by PCR. Genomic DNA was extracted from transgenic *A. thaliana* leaves and the *S-hyIgA* genes were amplified by PCR using gene specific primers. *ACTIN2 (ACT2*) is an internal control gene of *A. thaliana*. (**c**) Transcription of *S-hyIgA* transgenes in the plant leaves. The mRNAs in the transgenic or wild type *A. thaliana* leaves were reverse transcribed, and then cDNAs of *H, L, J, SC* and *ACT2* were amplified by PCR. Secretory Tg, transgenic *A. thaliana*; WT, wild-type *A. thaliana*.

A binary vector was constructed for the expression of a plant-based monoclonal secretory hybrid-IgG/IgA (S-hyIgA) specific for Stx1. S-hyIgA consists of immunoglobulin heavy and light chains, a joining (J) chain, as well as a secretory component (SC), which is a product of mucosal epithelial cells. The S-hyIgA expression vector is formed from the pBCH1 binary vector^27^ harbouring the hybrid-IgG/IgA *heavy (H)* and *light (L) chain* expression cassette, and the *J chain* and *SC* expression cassette (Fig. 1a). The *H chain, L chain, J chain* and *SC* genes were expressed under the control of a bidirectional *CAB* promoter and terminator derived from *A. thaliana* (P_*CAB*_, T_*CAB1*_ and T_*CAB2*_). The S-hyIgA expression binary vector was introduced into *A. thaliana* through *Agrobacterium*-mediated transformation. After selection with hygromycin B, we obtained 12 transgenic lines. Leaf extracts of the transgenic lines were screened for the IgA protein expression level by means of sandwich ELISA, in which anti-κ was used for capture and anti-α for detection. IgA levels in each leaf extract were compared by the absorbance readings relative to that of the highest IgA-producing line SH4 at 100 μg/mL of total soluble protein (TSP). Among 12 lines, nine lines produced IgA with more than 50% of the absorbance reading of SH4, while three lines produced less than 5%. The mean and SD were 62 ± 38% (n = 12). The line exhibiting the highest IgA expression was used after obtaining homozygote by self-pollination.

The genes that are the parts of the *S-hyIgA* genes (*H, L* and *J chains* and *SC*) were amplified by PCR using genomic DNA from transgenic *A. thaliana* (secretory Tg) leaves. No such PCR fragment was amplified from leaves of wild type plants. A housekeeping gene, *ACT2*, was equally detected in both transgenic and wild type leaves (Fig. 1b). In agreement with this, mRNA transcripts of *S-hyIgA* genes were detected in total RNA extracts from only secretory Tg leaves on RT-PCR (Fig. 1c). Transcripts of *ACT2* from secretory Tg and wild-type leaves were equally detected.

### Expression of S-hyIgA proteins in transgenic *A. thaliana* plant leaves

**Figure 2:**
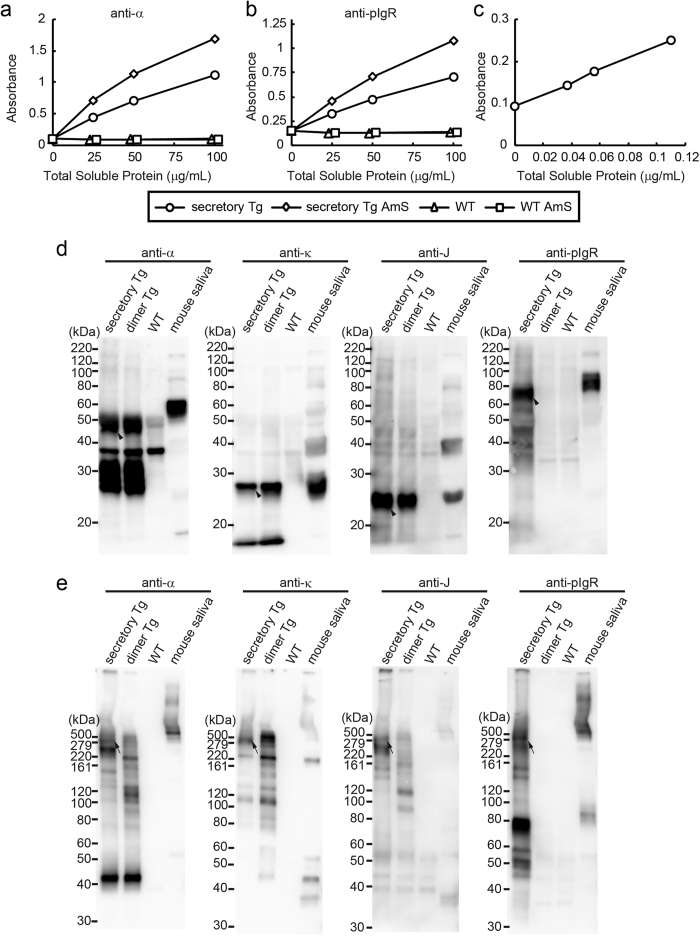
Expression of S-hyIgA proteins in *A. thaliana*. (**a,b**) Detection of assembled hybrid-IgG/IgA by sandwich ELISA. The hybrid-IgG/IgA proteins in each sample were captured with immobilized anti-κ antibodies and detected with anti-α (**a**) or anti-pIgR (**b**) antibodies. Crude leaf extracts and 50%-saturated ammonium sulphate (AmS)-concentrated ones were compared. (**c**) Detection of SC protein by sandwich ELISA. The SC protein in the crude leaf extracts was captured with goat anti-pIgR and detected with rabbit anti-pIgR antibodies. The symbols represent crude extracts from secretory Tg (open circles) or WT plants (open triangles), or represent AmS-concentrated ones from secretory Tg (open diamonds) or WT plants (open squares). Data are expressed as means of triplicate determinations. All error bars (SD) are shorter than the size of symbols. The results are representative of four experiments. (**d,e**) Immunoblot analysis of S-hyIgA expressed in transgenic *A. thaliana* leaves. Leaf proteins precipitated with 50%-saturated ammonium sulphate were separated by SDS-PAGE under reducing (**d**; 12% gel, 15 μg protein/lane) or non-reducing (**e**; 4–20% gradient gel, 5 μg protein/lane) conditions, and then blotted onto a PVDF membrane. Each polypeptide chain of secretory IgA was detected with anti-α, anti-κ, anti-J and anti-pIgR antibodies. Secretory Tg (quadruple transgenic for H, L, J and SC), dimer Tg (triple transgenic for H, L and J), and WT (wild-type) *A. thaliana*, and mouse saliva (secretory IgA positive control) were analysed.

Total soluble proteins (TSP) were extracted from secretory Tg leaves and then hybrid-IgG/IgA protein in the extract was quantitated by ELISA (Fig. 2a). The hybrid-IgG/IgA was captured with an immobilized anti-κ antibody, followed by detection with an anti-α antibody. The signals representing antibodies with both H and L chains increased with increasing TSP in the crude extract from secretory Tg leaves (open circles). In contrast, no signal was detected for wild type leaves (open triangles). The hybrid-IgG/IgA concentration was calculated by comparison with IgA myeloma TEPC 15 as a standard. The production volume of assembled hybrid-IgG/IgA reached 8.0 μg/g leaf tissue (0.07% of TSP). Because SC consists of the extracellular domains of pIgR, we used anti-pIgR antibodies to detect SC. The S-hyIgA in the secretory Tg leaf extract was detectable with anti-pIgR antibodies (Fig. 2b). The S-hyIgA-specific signals also increased with increasing TSP in the secretory Tg sample (open circles). No such signal was detected for the wild type leaves (open triangles). To measure the total SC concentration, sandwich ELISA was performed using goat anti-pIgR as a capture antibody and rabbit anti-pIgR as a detection antibody. The signals representing SC increased with increasing TSP from secretory Tg leaves (Fig. 2c). Compared with a standard curve generated with recombinant pIgR, the total SC concentration was calculated. The production volume of the total SC reached 57.7 μg/g leaf tissue (0.31% of TSP).

Proteins were concentrated from the leaf extracts by ammonium sulphate (AmS) precipitation. When concentrated leaf samples were examined (open diamonds), approximately two-fold enrichment was observed for both IgA (Fig. 2a) and SIgA (Fig. 2b) by comparing with the crude extract (open circles). No signal was detected in the wild type leaf concentrate (open squares).

The presence of each component of S-hyIgA in the concentrate was investigated by SDS-PAGE and immunoblotting using specific antibodies. In this experiment, we also performed comparison with the dimer hybrid-IgG/IgA transgenic *A. thaliana* (dimer Tg) expressing H, L and J chains but no SC, which had been established previously^25^. Under reducing conditions, α, κ and J chains were detected in the secretory Tg as well as in the dimer Tg^25^, whereas SC was only detected in the secretory Tg (Fig. 2d, arrowheads). The bands representing the α chain and SC were observed around 50 kDa and 70 kDa, respectively, for the transgenic plants. In contrast, secretory IgA in mouse saliva exhibited values of 60 kDa and 90 kDa, respectively, for the α chain and SC. Some additional bands were observed by the anti-α chain (around 30 kDa) and anti-κ chain (under 20 kDa) in the secretory Tg and dimer Tg leaves, but we did not carry out further identification of these bands.

Under non-reducing conditions, bands corresponding to the assembled S-hyIgA were detected with antibodies specific for each immunoglobulin chain in the secretory Tg leaves. (Fig. 2e, arrows). These bands corresponded to smaller molecular masses than that of secretory IgA from mouse saliva. Some other bands were seen for the secretory Tg sample. These bands may represent the H chain dimer with a J chain and an SC, free SC and their fragments. The secretory Tg but not the dimer Tg sample gave bands detectable with anti-pIgR. These results indicate that S-hyIgA is expressed and assembled in the secretory Tg plant leaves.

### Subcellular localization of hybrid-IgG/IgA in leaf tissue of transgenic *A. thaliana*

**Figure 3:**
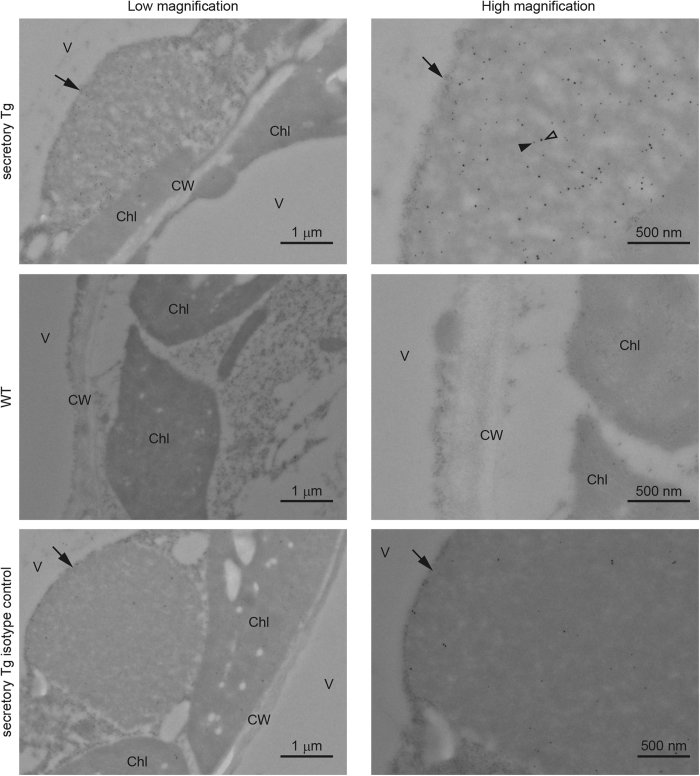
Immuno-electron microscopic analysis of leaf tissue of transgenic *A. thaliana*. Transverse sections of *A. thaliana* leaf tissue were stained with anti-α chain (18-nm gold label, open arrowhead) or anti-pIgR (12-nm gold label, closed arrowhead) antibodies. Arrows indicate intracellular protein body-like structures containing S-hyIgA. V, vacuole; Chl, chloroplast; CW, cell wall. Bars represent 1 μm (low magnification) or 500 nm (high magnification).

To determine the intracellular localization of S-hyIgA in plant cells, we prepared ultrathin sections of leaf tissue and analysed them by means of immuno-electron microscopy using antibody-conjugated colloidal gold particles (Fig. 3). A protein body-like structure was observed in the transgenic plant cells (arrows). Co-localization of the colloidal gold-labelling signals representing the α chain (18 nm-particle, open arrowhead) as well as SC (12 nm-particle, closed arrowhead) was observed within the protein body-like structure. The diameter of the protein body-like structures approximately ranged from 1 to 6 μm. Such a structure was not seen in the wild type plant cells. In the isotype controls, colloidal gold-labelling signals were scarcely observed in the protein body-like structure.

### Glycosylation of plantibodies

**Figure 4:**
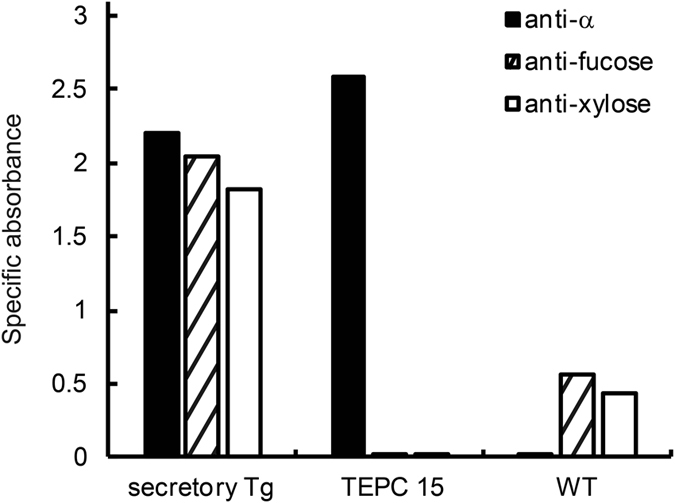
Plant-type glycosylation on the S-hyIgA. Plant-specific carbohydrate moieties were detected by sandwich ELISA. Antibodies in leaf protein extracts were allowed to be captured with immobilized anti-κ antibodies, and then detected with antibodies against IgA heavy chain (filled columns), ones against Fuc α(1,3) GlcNAc (hatched columns) or ones against xylose β(1,2) Man (open columns). The signals from wells without coating of anti-κ antibodies had been subtracted. Leaf proteins from secretory Tg (IgA: 1 μg/mL, TSP: 0.6 mg/mL) or those from wild-type (WT; TSP 0.6 mg/mL) as well as mouse IgA myeloma protein TEPC 15 (IgA 1 μg/mL) were tested. Data are expressed as means of triplicate determinations, and SD does not exceed 6% of the mean under each condition.

Protein bodies are classified into endoplasmic reticulum (ER)-derived protein body-I (PB-I) and protein storage vacuole protein body-II (PSV or PB-II)^28^. Glycoproteins accumulated in the PSV are believed to have undergone sugar chain processing in the Golgi apparatus in plant cells^29^. Thus, it is useful to determine whether S-hyIgA has undergone plant-specific modification of sugar chains for classifying S-hyIgA accumulated in the protein body-like structure. Two types of modifications associated with the core of *N*-glycans were examined. One was Fuc α(1,3) GlcNAc and the other xylose β(1,2) Man. Plantibodies were captured with immobilized anti-κ antibodies and then detected with appropriate detection antibodies on ELISA. Because of the relatively high background for plant samples due to non-specific adsorption on ELISA wells, the signals from wells with BSA blocking only were subtracted from those from wells coated with anti-κ antibodies. Successful capture of S-hyIgA was demonstrated by detection with anti-α antibodies. Samples from secretory Tg as well as mouse IgA myeloma TEPC 15 proteins produced α chain-specific signals, whereas samples from wild type plants did not. Samples from secretory Tg leaves exhibited the binding of antibodies specific for α1,3-linked Fuc as well as one for β1,2-linked Xyl (Fig. 4). TEPC 15 did not show plant-specific modifications, consistent with it being a mouse protein. In wild type plants, signals of plant-type glycosylation remained, but the signal levels were lower than those for transgenic plants. We concluded that the S-hyIgA was equipped with plant-type sugar chains, because the signals for plant-specific glycosylation were shown to be dependent on anti-κ capture.

### The S-hyIgA binds to Stx1B

**Figure 5:**
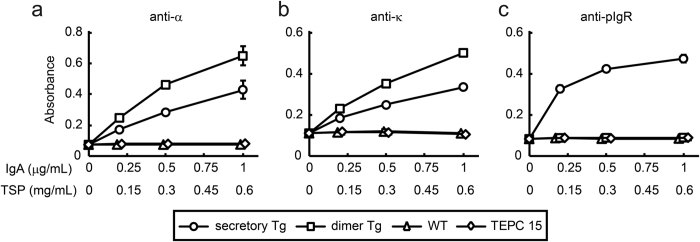
Binding of S-hyIgA to immobilized Stx1B. Leaf extract samples (50%-saturated ammonium sulphate precipitation fraction) from secretory Tg (open circles), dimer Tg (open squares), or wild-type (open triangles) plants, or purified mouse IgA myeloma protein TEPC 15 (open diamonds) were allowed to bind to immobilized Stx1B. The bound antibodies were detected with anti-α (**a**), anti-κ (**b**), or anti-pIgR (**c**) antibodies. Data are expressed as means ± SD of triplicate determinations. Error bars underneath the symbols are not visible. For comparison, the results for secretory Tg and dimer Tg leaves, and TEPC 15 are plotted against IgA concentration, while those for secretory Tg and wild-type leaves against TSP, on the abscissa. The results are representative of three experiments.

We then investigated whether the S-hyIgA plantibodies bound to Stx1B by means of ELISA. Anti-α as well as anti-κ second antibodies revealed dose dependent-binding of the plantibodies from the secretory Tg as well as dimer Tg leaves (Fig. 5a and b). Anti-pIgR antibodies only detected the binding of the secretory plantibodies from the secretory Tg leaves, while no signal was seen for the dimer Tg leaves (Fig. 5c). Stx1B-specific antibody binding was not observed using wild type leaf samples or TEPC 15. These results demonstrated that Stx1B-specific S-hyIgA is produced by the secretory Tg but not by dimer Tg leaves.

### Neutralization of Stx1-mediated cytotoxicity by plantibody

**Figure 6:**
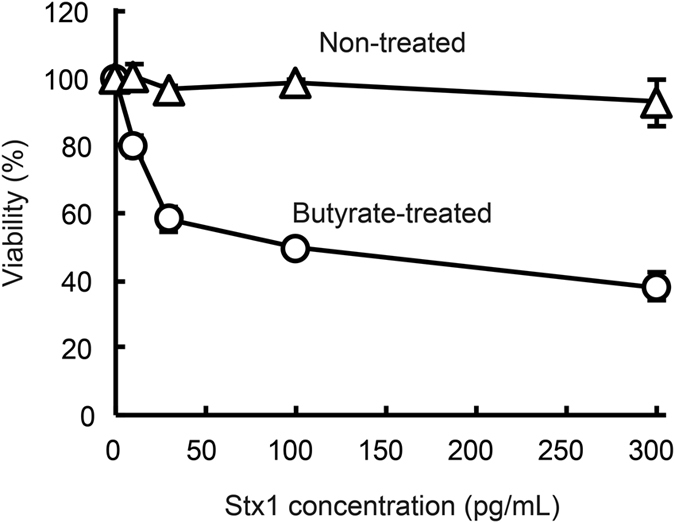
Butyrate treatment increased the sensitivity of Caco-2 cells to Stx1. Caco-2 cells were cultured with 2 mM sodium butyrate-containing medium or normal medium for 4 days. The cells were then exposed to various concentrations of Stx1 (0, 10, 30, 100 or 300 pg/mL) for 48 h. The viable cells were quantitated by means of WST-8 cell viability assaying. Data for butyrate-treated cells (open circles) and untreated cells (open triangles) are shown. Viability was calculated as the relative value to that without toxin exposure. Data are expressed as means ± SD of triplicate determinations. Error bars underneath the symbols are not visible.

Toxin neutralization by plantibodies was then examined. We first used a human colonic epithelial cell line, Caco-2, to assess Stx1-induced cytotoxicity. We think that direct cytotoxicity of Stx1 toward colonic epithelial cells may initiate food poisoning. Caco-2 cells are known to upregulate cell surface expression of Gb_3_, the target glycolipid for Stx1, on treatment with butyrate^22^. Under our current experimental conditions, Caco-2 cells became susceptible to Stx1 after butyrate treatment for 4 days. Thus, the viability of butyrate-treated Caco-2 cells decreased after 48 h exposure to Stx1 in a dose-dependent manner, whereas that of untreated Caco-2 cells did not (Fig. 6).

**Figure 7:**
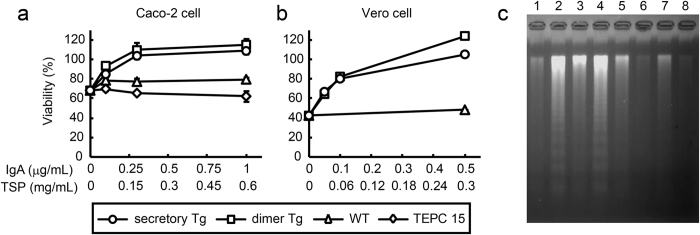
Neutralization of Stx1 cytotoxicity with S-hyIgA plantibodies *in vitro*. Stx1 holotoxin (10 or 100 pg/mL) was pre-incubated with a sample from secretory Tg plants, dimer Tg plants, or wild-type plants (50%-saturated ammonium sulphate-precipitated preparation of leaf extract), or with purified mouse IgA myeloma protein TEPC 15. The mixture was added to butyrate-treated Caco-2 cells (Stx1 100 pg/mL) or untreated Vero cells (Stx1 10 pg/mL). After cells had been cultured for 48 h, the cytotoxicity of Stx1 was evaluated by the following assays. (**a,b**) Viability of Caco-2 cells (**a**) or Vero cells (**b**) was determined by WST-8 assaying. The symbols represent secretory Tg plants (open circles), dimer Tg plants (open squares), wild-type plants (open triangles), or IgA myeloma TEPC 15 (open diamonds). The viability (ordinate) is relative to that of toxin untreated cells. Data are expressed as means ± SD of triplicate determinations. Error bars underneath the symbols are not visible. The doses of antibodies are expressed as IgA concentration or TSP (abscissa), as shown in Fig. 5(c). Apoptosis of butyrate-treated Caco-2 cells was detected as DNA fragmentation. After exposure to Stx1 (100 pg/mL), the DNA fragmentation was visualized by agarose gel electrophoresis. Lane 1, untreated; lane 2, Stx1-treated; lane 3, Stx1 + WT-treated (TSP 0.6 mg/mL); lane 4, Stx1 + TEPC 15-treated (1 μg/mL IgA); lane 5, Stx1 + secretory Tg (0.1 μg/mL IgA); lane 6, Stx1 + secretory Tg (1 μg/mL IgA); lane 7, Stx1 + dimer Tg (0.1 μg/mL IgA); and lane 8, Stx1 + dimer Tg (1 μg/mL IgA).

Butyrate-treated Caco-2 cells were used as a target to determine Stx1 neutralization. The reduced viability caused by exposure to 100 pg/mL Stx1 was reversed in the presence of increasing concentrations of the plantibody sample from secretory Tg or dimer Tg leaves (Fig. 7a). The reduced viability was not reversed in the presence of a wild type leaf sample or TEPC 15.

Because Vero cells are highly sensitive to Stx1 toxicity, we used them as another target. The reduced viability caused by exposure to 10 pg/mL Stx1 was reversed in the presence of increasing concentrations of a sample from secretory Tg or dimer Tg leaves, but not from wild type plants (Fig. 7b).

Because Stx1 is known to induce apoptosis of sensitive cells^30^,^31^, Stx1-induced apoptosis of butyrate-treated Caco-2 cells was assessed by means of DNA fragmentation assaying. The genomic DNA of Caco-2 cells became fragmented into length of multiples of 180 bp after 48 h exposure to 100 pg/mL Stx1 (Fig. 7c, lane 2). High molecular weight DNA did not enter the agarose gel, and was not visible for the Stx1-untreated cells (lane 1). When Stx1 was pre-incubated with plantibodies, the DNA fragmentation of Caco-2 cells was inhibited (lanes 5 to 8). One μg/mL of plantibodies from secretory Tg (lane 6) or dimer Tg (lane 8) leaves completely prevented the DNA fragmentation induced by 100 pg/mL Stx1. Samples from wild-type leaves and TEPC 15 did not prevent DNA fragmentation (lanes 3 and 4).

In conclusion, a functional secretory IgA can be produced in transgenic *A. thaliana* using a promoter of plant origin. The *in vitro* neutralization capacities were similar between samples from secetory Tg and dimer Tg plants on the basis of the IgA concentrations.

## Discussion

Plant expression systems are useful tools for the production of oral immunotherapeutic agents including SIgA because of easy administration, i.e., eating edible host plants^13^. We previously established a hybrid-IgG/IgA consisting of mouse monoclonal IgG against Stx1B and mouse IgA constant regions^16^. To produce SIgA that can neutralize Stx1 toxicity, we established transgenic plants expressing S-hyIgA by means of the *Agrobacterium*-method.

Viral promoters such as the cauliflower mosaic virus *35S* promoter are often used in plant expression system. However, it will be useful to test whether an alternative expression system works without using virus-derived DNA elements with respect to public acceptance. We chose the *Arabidopsis*-derived *CAB* promoter to express S-hyIgA (Fig. 1a). The *CAB* promoter is known to allow a high level of expression as well as bi-directional transcription. Thus, this promoter is suitable for the co-expression of two proteins in leaf tissue^26^. Regarding the protein expression levels, the SC protein was expressed at a high level (57.7 μg/g leaf tissue). This expression level is comparable to that in other reports describing stable expression of therapeutic proteins in plants^32^,^33^.

On the other hand, the expression level of assembled hybrid-IgG/IgA that has both heavy and light chains was 8.0 μg/g leaf tissue, which was lower than in other reports for stable expression of IgA in plants^34^. Proteolytic degradation of heavy chains is considered as one of the reasons why the hybrid-IgG/IgA was expressed at a low level in contrast to the high level of SC expression. On western blot analysis, a large amount of degraded heavy chains was observed around 20~30 kDa (Fig. 2d). Some reports described that the hinge region of a human IgG plantibody has a weak spot as to proteolysis in plant cells^35^,^36^,^37^. The hinge region of the hybrid-IgG/IgA is derived from that of mouse IgG, and the amino acid sequence around the weak spot is similar to that reported, that is KDVKKIVP in our construct and KDVKKVEP in other reports. Replacement of the hinge region may improve hybrid-IgG/IgA expression in plant cells.

Intracellular localization of S-hyIgA is a key factor as to protein yield and stability. Translated S-hyIgA was accumulated in a protein body-like structure, which was observed only in transgenic plants (Fig. 3). A protein body-like structure was also reported in other transgenic plant leaves^38^. Plant-specific glycosylation on S-hyIgA (Fig. 4) suggested that S-hyIgA was accumulated in a PSV-like vacuole. Storage proteins in PSV are known to be transported from the Golgi apparatus, in which plant-specific glycosylation occurs^29^. PSV localization of plant-expressed IgA was also reported in rice^39^. PSV functions to supply amino acids for plant seed development through degradation of storage proteins^40^. Thus, a high rate of IgA degradation may be due to the localization in this structure. Modification of intracellular localization of S-hyIgA may increase its yield.

The EHEC infection is difficult to treat because a large amount of Stx is released from bacteria upon treatment with conventional antibiotics. Oral passive immunotherapy is a candidate treatment for EHEC infection as an alternative one to antibiotics. Despite that life-threatening symptoms are dependent on the entry of Stx into the blood circulation, it is still controversial as to how Stx initially enters the blood^41^.

It is natural to consider that Stx enters due to a breach in the intestinal epithelial barrier. However, it is not clear whether Stx-induced damage to intestinal epithelial cells is responsible for the breach. One hypothesis is as follows: local inflammation due to the EHEC infection induces transmigration of neutrophils through the intestinal epithelia. The neutrophil transmigration triggers defects in the intestinal barrier that allow passive transfer of Stx into the bloodstream. A monolayer of human colon carcinoma T84 cells was used to demonstrate neutrophil transmigration in the presence of inflammatory cytokines^42^. T84 cells lack Gb_3_ and are resistant to the direct cytotoxicity of Stx. Despite this, the translocation of Stx was reported to be enhanced with neutrophil transmigration using a polarized T84 monolayer system. In a mouse system, we demonstrated that the binding sites for Stx1B as well as Gb_3_ expression were restricted to the distal part of the colon^43^. Furthermore, apoptosis of epithelial cells isolated from the distal but not from proximal mouse colon was observed upon exposure to Stx1^44^.

With this background, we used human colon epithelial Caco-2 cells to measure Stx1 cytotoxicity in this study. The cytotoxicity was enhanced after butyrate treatment (Fig. 6), which is consistent with an increase in Gb_3_ on butyrate-treated Caco-2 cells^22^. The leaf samples from S-hyIgA transgenic plants inhibited Stx1 cytotoxicity not only against Vero cells but also against butyrate-treated Caco-2 cells (Fig. 7). Stx1-iduced apoptosis of Caco-2 cells was also inhibited by the leaf samples from S-hyIgA transgenic plants. These results indicated that Stx1 is cytotoxic to human colon epithelial cells under certain circumstances. Stx1-sensitive human colon cells were protected by a plantibody specific for Stx1B. We already observed that the current construct is applicable to the expression of S-hyIgA in lettuce leaves (manuscript in preparation). Continuous efforts to increase production volume will provide a key for success toward practical edible IgA therapeutics.

We have previously established *A. thaliana* producing dimeric hyIgA where IgA heavy and light chains are expressed under a *CAB* promoter while J chain is expressed under a *35S* promoter^25^. In contrast, the current S-hyIgA is expressed only using two *CAB* promoters in the absence of *35S* promoter. Despite this difference, the production volume of S-hyIgA was 8.0 μg/g fresh weight and that of dimeric hyIgA was 11 μg/g^25^. Although directing to other antigens, other studies revealed that SIgA production was 15.2 μg/g in transgenic Tobaccos^11^ and that transient *Nicotiana benthamiana* produced SIgA ranging from 25 μg/g to 32.5 μg/g^11^,^12^. The *in vitro* neutralization activities of our S-hyIgA and dimeric hyIgA against Stx1 were similar based on IgA concentrations. This was the case regardless of whether differentiated Caco-2 colonic cell or highly Stx1 sensitive Vero cells were used. A comparison between dimeric hyIgA and S-hyIgA will be best made by examination of *in vivo* efficacy and protease resistance. We are planning to test these questions.

In conclusion, we succeeded in producing a transgenic *A. thaliana* plant expressing Stx1B-specific hybrid-IgG/IgA as a secretory form. All four genes for coding antibody components were expressed without using a viral promoter. The plant-based S-hyIgA bound to Stx1B, and the leaf extract of transgenic plants successfully neutralized the cytotoxicity of Stx1 toward human colon epithelial cells, which represent Stx1 entry sites into the human bodies. Through further improvement of the protein yield and stability in plant cells, transgenic plants expressing S-hyIgA will become an effective tool for oral passive immunotherapy against food poisoning caused by Stx1. In addition, S-hyIgA will become widely applicable for the treatment of other mucosal infections by replacing the variable regions.

## Materials and Methods

### Construction of a secretory hybrid-IgG/IgA expression vector

We previously constructed a hybrid-IgG/IgA expression cassette for the expression of antibody heavy and light chains in plants^25^. To express J chain and SC proteins, a Jc-SC expression cassette was constructed as follows: mouse J chain cDNA^16^ was amplified by PCR using J chain-specific primers named JCF15 and JCR-*Xho* (Table 1). The amplified cDNA was digested with *Xba*I and subcloned into the *Xba*I/*Eco*RV-digested pcDNA3.1 vector (Invitrogen, Carlsbad, CA, USA). The subcloned J chain cDNA was digested with *Xba*I/*Xho*I and ligated into the pGEM5zf vector (Promega, Madison, WI, USA) harbouring chlorophyll *a/b*-binding protein terminator 2 (T_*CAB2*_)^25^, resulting in the production of pGEM5zf/Jc-T_*CAB2*_. To subclone mouse SC cDNA, PCR was performed using pcDNA3.1/mouse SC^45^ as a template, and SC-specific SCF-*Xho* and SCR-*Xba* as primers. Following *Xba*I/*Xho*I digestion and generation of blunt ends, SC cDNA was inserted into *Eco*RV-digested pGEM5zf harbouring T_*CAB1*_(pGEM5zf/T_*CAB1*_), resulting in the production of pGEM5zf/SC-T_*CAB1*_. pGEM5zf/Jc-T_*CAB2*_ and pGEM5zf/SC-T_*CAB1*_ were digested with *Sac*II/*Hin*dIII. The resulting DNA fragments, Jc-T_*CAB2*_ and SC-T_*CAB1*_, were ligated with *Hin*dIII-digested pUC19 (Invitrogen) by means of three-piece ligation, resulting in the production of pUC19/T_*CAB2*_-Jc-SC-T_*CAB1*_. pUC19/T_*CAB2*_-Jc-SC-T_*CAB1*_ was digested with *Sac*II and then ligated with *Sac*II-digested P_*CAB*_, resulting in the production of the pUC19/Jc-SC expression cassette. The Jc-SC expression cassette was inserted into the pBCH1 binary vector^27^ in two steps. First step was the insertion of T_*CAB2*_ into pBCH1. The pGEM5zf/T_*CAB2*_ was digested with *Hin*dIII and then blunt ends were generated with the Klenow fragment (Takara Bio, Shiga, Japan). The blunt-ended DNA was digested with *Sac*I and ligated into *Sma*I/*Sac*I-digested pBCH1, resulting in the production of pBCH1/T_*CAB2*_. The second step was insertion of the Jc-P_*CAB*_-SC-T_*CAB1*_ to pBCH1/T_*CAB2*_. The pUC19/Jc-SC expression cassette was digested with *Hin*dIII/*Sac*I to obtain Jc-P_*CAB*_-SC-T_*CAB1*_ DNA fragments, and then ligated into *Hin*dIII/*Sac*I-digested pBCH1/T_*CAB2*_, resulting in production of the pBCH1/Jc-SC expression cassette. During this process, the cauliflower mosaic virus *35S* promoter was deleted from the pBCH1 vector. The pBCH1/Jc-SC cassette was digested with *Hin*dIII and ligated with the *Hin*dIII-digested hybrid-IgG/IgA expression cassette, resulting in the production of pBCH1/secretory hybrid-IgG/IgA.

### Transformation of *Arabidopsis thaliana*

Transformation and selection of *A. thaliana* was performed as previously described^25^. As a binary vector, pBCH1/secretory hybrid-IgG/IgA was used. Homozygous transformants were obtained by self-pollination.

### DNA analysis of transgenic *A. thaliana* by PCR

To confirm the S-hyIgA transgenes in the transgenic *A. thaliana* genomes, PCR was performed as previously described^25^ with the gene-specific primer sets (Table 1).

### mRNA analysis of transgenic *A. thaliana* by RT-PCR

Total RNA was purified from the transgenic plant (4-wk old) leaves using an RNeasy Mini Kit (QIAGEN, Hilden, Germany). Purified RNA samples were analysed by RT-PCR using an AccessQuick RT-PCR System (Promega) and the same gene-specific primer sets as for DNA analysis.

### Protein extraction and ammonium sulphate precipitation

Leaves from the transgenic plants (4-wk old) were frozen and crushed in liquid nitrogen. A protein extraction buffer (50 mM acetate buffer [pH 5.0], 500 mM NaCl, 0.5 mM EDTA, protease inhibitor cocktail for plant cell and tissue extracts [Sigma-Aldrich, St. Louis, MO, USA]) was added to each leaf sample. The leaf tissue suspension was centrifuged for 10 min at 15,000 × g at 4 °C. The supernatant was collected and neutralized with 1 M Tris-HCl (pH 8.0). After centrifugation, ammonium sulphate was added to the supernatant to 50% saturation on ice, and proteins were allowed to precipitate overnight at 4 °C. A sample was centrifuged for 10 min at 15,000 × g at 4 °C, and the precipitate was re-dissolved in PBS. Remaining insoluble aggregates were removed by centrifugation for 10 min at 21,500 × g at 4 °C. The supernatant was dialyzed against PBS with Mini Dialysis Kit 8 kDa cut-off (GE Healthcare, Buckinghamshire, UK). The resulting sample was used as the 50%-saturated ammonium sulphate precipitated fraction.

### ELISA

To determine the hybrid-IgG/IgA in samples, a sandwich ELISA was performed as previously described^16^. To measure total hybrid-IgG/IgA, goat anti-mouse κ (1 μg/mL; SouthernBiotech, Birmingham, AL, USA) was used as a capture antibody and horseradish peroxidase (HRP)-goat anti-mouse IgA (1:1,000 dilution; SouthernBiotech) as a detection antibody. To block non-specific binding, phosphate-buffered saline (PBS) containing 1% bovine serum albumin (BSA) was used. To measure S-hyIgA, the capture antibody was goat anti-mouse κ, but rabbit anti-mouse pIgR (0.5 μg/mL; Sino Biological, Beijing, China) was used as the detection primary antibody. To measure total SC, goat anti-mouse pIgR (1 μg/mL; R&D Systems, Minneapolis, MN, USA) was used as a capture antibody and the detection primary antibody was rabbit anti-mouse pIgR. As standards, TEPC 15 mouse IgA myeloma proteins were purchased from Sigma-Aldrich, and recombinant soluble mouse pIgR (extracellular domains) from R&D Systems. To detect plant-specific sugar chains on hybrid-IgG/IgA, the same capture antibody was used for total hybrid-IgG/IgA and rabbit anti-α1,3-fucose (0.5 μg/mL; Agrisera, Vännäs, Sweden) or rabbit anti-β1,2 xylose (1 μg/mL; Agrisera) were used as detection primary antibodies. To eliminate the effects of non-specific adsorption of plant proteins to ELISA wells, signals from wells with BSA-blocking alone were subtracted from those with anti-κ coating. The binding activity of hybrid-IgG/IgA to immobilized Stx1B was analysed by means of ELISA^16^. As detection primary antibodies, HRP-goat anti-mouse IgA (1:1,000 dilution), goat anti-mouse κ (1 μg/mL) or rabbit anti-mouse pIgR (0.5 μg/mL) were used. For all indirect ELISA, HRP-goat anti-rabbit IgG (1:1,000 dilution; SouthernBiotech) or HRP-donkey anti-goat IgG (0.5 μg/ml; Santa Cruz Biotechnology, Santa Cruz, CA, USA) were used as the secondary antibodies.

### SDS-PAGE and immunoblotting

The hybrid-IgG/IgA protein in the 50%-saturated ammonium sulphate precipitation sample was analysed by SDS-PAGE and immunoblotting as described previously^16^. Proteins were separated under reducing (12% Mini-PROTEAN TXG Gel, Bio-Rad) or non-reducing (4–20% Mini-PROTEAN TXG Gel, Bio-Rad) conditions, and blotted on PVDF membranes (Bio-Rad). The hybrid-IgG/IgA was detected with HRP-goat anti-mouse IgA (1:1,000 dilution; SouthernBiotech), goat anti-mouse κ (0.2 μg/mL; SouthernBiotech) plus HRP-donkey anti-goat IgG (0.2 μg/mL; Santa Cruz), rabbit anti-mouse J chain (0.4 μg/mL; Santa Cruz) plus HRP-goat anti-rabbit IgG (1:3,000 dilution; SouthernBiotech) or rabbit anti-mouse pIgR (0.2 μg/mL; Sino Biological) plus HRP-goat anti-rabbit IgG (1:3,000 dilution). HiMark^TM^ Pre-Stained High Molecular Weight Protein Standards (Invitrogen) and MagicMark^TM^ XP Western Protein Standards (Invitrogen) were used as molecular weight standards.

### Immuno-electron microscopy

The leaf tissue of *Arabidopsis* plants was fixed in 2% (v/v) glutaraldehyde-0.1 M potassium phosphate buffer (pH7.2) and embedded in LR White (London Resin, Berkshire, UK). Ultrathin sections (100 nm) were blocked with 5% BSA-PBS and then labelled with goat anti-mouse pIgR (1 μg/mL; R&D Systems) and rabbit anti-mouse IgA (1:1,000; Abcam, Cambridge, UK). Normal goat IgG and normal rabbit IgG were used as isotype controls. The bound primary antibodies were detected with 12 nm colloidal gold-donkey anti-goat IgG (1:100 dilution; Jackson ImmunoResearch, West Grove, PA, USA) and 18 nm colloidal gold-donkey anti-rabbit IgG (1:100 dilution; Jackson ImmunoResearch). After electron staining, the sections were examined under a JEM-1011 transmission electron microscope (JEOL, Tokyo, Japan) at 80 kV.

### Cell viability assay

To measure the cytotoxicity of Stx1 and neutralization by plantibodies, WST-8 cell viability assaying was performed as previously described^25^. To compare the Stx1-sensitivity of Caco-2 cells with or without butyrate treatment, Caco-2 cells were seeded at 2 × 10^4^ cells/well in a 96-well cell culture plate (Falcon^®^ 353072) and cultured for 3 days in 10% foetal bovine serum (FBS; Hyclone, South Logan, UT, USA)-Dulbecco’s modified Eagle’s medium (DMEM; Nissui Pharmaceuticals, Tokyo, Japan) supplemented with 1% non-essential amino acids (GIBCO BRL, Palo Alto, CA) at 37 °C under a humidified atmosphere of 5% CO_2_/95% air. The cells were treated with 2 mM sodium butyrate in the medium for 4 days to increase Gb_3_ expression on the cell membrane. The butyrate untreated cells were continuously cultured in the medium. Stx1 was added to Caco-2 cells at 0, 10, 30, 100 or 300 pg/mL. After 48 h culture, cell viability was assessed by WST-assay cell viability assaying (Cell Counting Kit-8; DOJINDO, Kumamoto, Japan) following the manufacturer’s instructions. Cell viability was calculated as the percentage of that of control level (no toxin exposure).

For toxin neutralization assaying using Caco-2 cells, 100 pg/mL of Stx1 holotoxin was incubated with the hybrid-IgG/IgA, an extract from wild-type plant leaves or mouse IgA myeloma protein TEPC 15 for 1 h at 37 °C. For plant preparation, proteins after precipitation with 50%-saturated ammonium sulphate were used. The mixture was then added to butyrate-treated Caco-2 cells. After 48 h culture, cell viability was assessed as above.

For toxin neutralization assaying using Vero cells, cells were seeded at 2 × 10^4^ cells/well in a 96-well cell culture plate and cultured for 16 h in 10% FBS-medium 199 (M199; GIBCO BRL). Preparation of the Stx1/plantibody mixture and Stx1 exposure were the same as in the experiments involving Caco-2 cells except that 10 pg/mL of Stx1 was used.

### DNA fragmentation assay

Caco-2 cells were cultured in a 24-well cell culture plate (Corning, Costar 3526, NY, USA) at 5 × 10^4^ cells/well for 3 days. Sodium butyrate treatment and Stx1 exposure were carried out as described above. After 48 h incubation with Stx1, genomic DNA was extracted, purified and separated by agarose gel electrophoresis. DNA fragmentation was visualized as described previously^31^.

## Additional Information

**How to cite this article**: Nakanishi, K. *et al*. Protection of Human Colon Cells from Shiga Toxin by Plant-based Recombinant Secretory IgA. *Sci. Rep.* **7**, 45843; doi: 10.1038/srep45843 (2017).

**Publisher's note:** Springer Nature remains neutral with regard to jurisdictional claims in published maps and institutional affiliations.

## Figures and Tables

**Table 1:** Primers used for PCR reactions.

Gene	Name of primers	DNA sequence
*J chain*	JCF15	5′-TCTTTCTAGAGTGAAGACAAGA-3′
*J chain*	JCR-*Xho*	5′-GTGCTGGATATCTCGAGAAT-3′
*J chain*	JCR	5′-CTAGTCAAGGTAGCAAGAAT-3′
*SC*	SCF-*Xho*	5′-CCTCTCGAGTCTCTTTAGTTGGCAAAAGGC-3′
*SC*	SCR-*Xba*	5′-CTTAATCTAGACAAGCAATGAGGCTCTACT-3′
*IgG H chain*	IgG Heavy *Not*F	5′-CACTGCGGCCGCTGACTCTAACCATGGGATGGAGC-3′
*IgA H chain*	IgA-H/*Not*R	5′-GGGCGGCCGCTCAGTAGCAGATGCCATCTCCCTC-3′
*IgG L chain*	IgGk *Not*F	5′-TGTGCGGCCGCAGCAGAAACATGAAG-3′
*IgG L chain*	IgGk *Not*R	5′-CGAGCGGCCGCTTCTAACACTCATTCC-3′
*ACTIN2*	actin2-F	5′-GTTGGTGATGAAGCACAATCCAAG-3′
*ACTIN2*	actin2-R	5′-CTGGAACAAGACTTCTGGGCATCT-3′
